# High Loss of Adipose Tissue During Neoadjuvant Chemotherapy Predicts Poor Prognosis in Patients With Gastric Cancer

**DOI:** 10.1002/jcsm.70107

**Published:** 2025-10-30

**Authors:** Chiyu He, Kankai Zhu, Qingqu Guo, Li Chen, Dan Wu, Lele Lin, Xiaoyong Zhang, Qianyun Shen, Weihuai Liu, Qi Zhang, Xinbao Wang, Ping Hu, Zhiqiang Zheng, Xianfa Wang, Zhilong Yan, Qing Zhang, Chunhui Shou, Yongqiang Si, Xingnan Wu, Tianzhe Gao, Yuan Gao, Jiren Yu, Xiaosun Liu

**Affiliations:** ^1^ Department of Gastrointestinal Surgery The First Affiliated Hospital, Zhejiang University School of Medicine Hangzhou China; ^2^ Department of Gastrointestinal Surgery The Second Affiliated Hospital, Zhejiang University School of Medicine Hangzhou China; ^3^ Hangzhou Institute of Medicine (HIM) Chinese Academy of Sciences Hangzhou China; ^4^ Department of General Surgery Beilun District People's Hospital Ningbo China; ^5^ Department of Gastrointestinal Surgery Zhejiang Provincial Hospital of Chinese Medicine Hangzhou China; ^6^ Department of Hepatopancreatobiliary Surgery Cancer Hospital of the University of Chinese Academy of Sciences Hangzhou China; ^7^ Department of General Surgery Lishui Central Hospital Lishui China; ^8^ Department of Gastrointestinal Surgery The Second Affiliated Hospital of Wenzhou Medical College Wenzhou China; ^9^ Department of General Surgery Sir Run Run Shaw Hospital, Zhejiang University School of Medicine Hangzhou China; ^10^ Department of Gastrointestinal Surgery Ningbo First Hospital Ningbo China; ^11^ Department of Gastrointestinal Surgery Yiwu Central Hospital Yiwu China

**Keywords:** adipose tissue, computer tomography, gastric cancer, muscle, neoadjuvant chemotherapy

## Abstract

**Background:**

Gastric cancer (GC) patients often have nutritional risks or malnutrition, and neoadjuvant chemotherapy (NAC) tends to exacerbate malnutrition. Body composition parameters are associated with the prognosis of GC patients. Little is known about body composition changes during NAC and its role in clinical outcomes.

**Methods:**

This was a secondary analysis of a Phase 3, open‐label, multicentre, randomized clinical trial (RCT) (NCT01364376), assessing the usefulness, safety and efficacy of S‐1 plus oxaliplatin (SOX) vs. fluorouracil, leucovorin and oxaliplatin (FOLFOX) as a perioperative chemotherapy regimen for patients with locally advanced GC. Pre‐NAC and post‐NAC computer tomography (CT) were collected to evaluate the prognostic role of skeletal muscle, adipose tissue and their dynamic change. Overall survival (OS) and progression‐free survival (PFS) rates were calculated using the Kaplan–Meier method.

**Results:**

A total of 583 patients from 12 Chinese hospitals were enrolled. After exclusion, 423 patients who proceeded to surgery were used for further analysis. The median age was 60 (IQR: 54, 66) years, and 133 patients (31.4%) were female. Prior to NAC, 132 (31.2%) patients were diagnosed with sarcopenia. There was no significant difference in overall survival between sarcopenia and non‐sarcopenia patients (*p* = 0.363). During NAC, most patients suffered skeletal muscle (SM) loss (*n* = 268, 63.4%), subcutaneous adipose tissue (SAT) loss (*n* = 236, 55.8%) and visceral adipose tissue (VAT) loss (*n* = 254, 60.0%). Patients with SAT loss > 12.5% had significantly worse PFS (*p* = 0.005) and OS (*p* = 0.003) than those without. Patients with VAT loss > 15% had significantly worse PFS (*p* = 0.003) and OS (*p* = 0.004) than the stable or gain group. Patients with SMI loss > 4.7% had significantly worse PFS (*p* = 0.043) and showed a tendency of reduced OS (*p* = 0.181) than those without. In patients without sarcopenia, the incidence of grade 3/4 neutropenia in the FOLFOX group was higher than that of the SOX group (*p* = 0.019). In patients with myosteatosis, the incidence of Grade 3/4 thrombocytopenia in the SOX group was higher than that of the FOLFOX group (*p* < 0.001).

**Conclusion:**

In this RCT study, patients with GC experience significant losses of muscle and adipose tissue during NAC. A high level of adipose tissue or muscle loss during NAC is prognostic of reduced survival in patients with locally advanced GC. Assessing and monitoring body composition can predict prognosis and effectively guide individual nutrition intervention and the prevention of complications.

## 1 Introduction

Gastric cancer (GC) is the fifth most common malignant tumour and one of the leading causes of cancer‐related death worldwide [1]. The most common symptoms of GC patients are related to digestion, including indigestion, anorexia or early satiety, weight loss and abdominal pain [2]. Therefore, GC patients often suffer from nutritional risks or malnutrition. Surgical resection remains a primary procedure for curative purposes. For resectable localized GC, neoadjuvant chemotherapy (NAC) is typically administered rather than upfront surgery followed by adjuvant therapy because of the fact that NAC could result in downstaging of a locally advanced tumour, address micrometastatic disease, improve survival and improve identification of patients who do not benefit from surgery due to disease progression during NAC [3, 4, 5, 6]. However, NAC often causes side effects and toxicity, which tend to exacerbate malnutrition. Therefore, it is of great significance to identify factors that might explain individual variation in therapeutic safety and efficacy.

Body composition has been studied in several types of tumours and has been suggested to be associated with cancer prognosis [7, 8, 9]. Cross‐sectional computed tomography (CT) imaging at the level of the third lumbar spine is considered as a standard approach to estimate the contents and distribution of skeletal muscle and adipose tissue due to its wide availability, high precision, and low incremental costs [10, 11]. According to the 2011 international consensus, cancer cachexia was defined as a multifactorial syndrome characterized by an ongoing loss of skeletal muscle mass (with or without loss of fat mass), which cannot be fully reversed by conventional nutritional support and leads to progressive functional impairment [12]. Cachexia is a common complication in gastric cancer and is often linked to poor chemotherapy response, decline in the quality of life and diminished survival [13, 14, 15, 16]. Sarcopenia, defined as the depletion of skeletal muscle mass and muscle dysfunction, is widely recognized to be associated with the prognosis of several types of tumours. Some recent research revealed that GC patients with sarcopenia had significantly shorter overall survival (OS) and more chemotherapy toxicity [17, 18, 19, 20]. Adipose tissue was another important component of body composition and was regarded as a marker for prognosis in GC patients [21, 22, 23]. Most studies found that patients with GC who receive NAC often have significant changes in body composition [24]. A recent study found that patients with foregut cancers experience significant losses of muscle during chemotherapy and a high level of skeletal muscle loss (> 6%) is prognostic of reduced survival [25]. In addition, muscle loss ≥ 5% was associated with a higher Mandard tumour regression grade in GC patients treated with pre‐operative fluorouracil plus leucovorin, oxaliplatin and docetaxel (FLOT) therapy [26]. However, the prognostic role of adipose tissue change is still unknown.

In 2022, we reported the results of the FOCUS trial, which compared the safety and efficacy of S‐1 plus oxaliplatin (SOX) vs. fluorouracil, leucovorin and oxaliplatin (FOLFOX) as a perioperative chemotherapy regimen for patients with locally advanced GC [27]. We concluded that SOX was non‐inferior to FOLFOX as perioperative chemotherapy for patients with GC. Herein, based on the data from the FOCUS trial, we assessed systematically the dynamic change of body composition parameters including skeletal muscle and adipose tissue during NAC and evaluated their prognostic role on survival outcomes and the association with relevant complications.

## 2 Methods

### 2.1 Design and Patients

**FIGURE 1:**
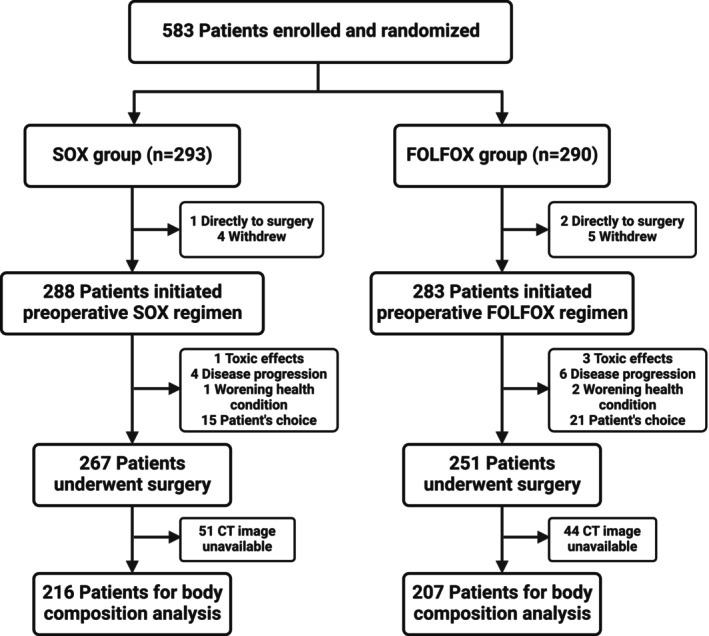
Flowchart of patient selection procedures. CT, computed tomography; FOLFOX, fluorouracil, leucovorin and oxaliplatin; SOX, S‐1 plus oxaliplatin.

We conducted a secondary analysis of the FOCUS trial, which was an investigator‐initiated, multicentre, open‐label randomized controlled trial involving 12 Chinese hospitals [27]. Briefly, patients with locally advanced gastric cancer were randomly assigned (1:1) to receive either perioperative FOLFOX or SOX. The inclusion and exclusion criteria were elaborated in our previous study [27]. SOX or FOLFOX was administered for 2–4 cycles as NAC, and surgery was scheduled within 2 weeks after completion of the last cycle of NAC. Patients who did not undergo surgery were excluded. Of these, CT scans of 423 patients (82%) were evaluable for our analysis of dynamic change of body composition parameters. The reason is related to the fact that the other patients' CT image data were lost. The flowchart of patient selection procedures was shown in Figure 1.

The study was done according to the Declaration of Helsinki and Good Clinical Practice Guidelines as defined by the International Conference on Harmonization. All enrolled patients provided written informed consent. The trial is registered at ClinicalTrials.gov (NCT01364376). The study protocol was approved by the ethics committees of participating institutions based on the ethical approval obtained from the ethics committee of the lead centre, the first affiliated hospital, Zhejiang University School of Medicine (approval number: ZDYYLS2024Y, No. 1105‐K).

### 2.2 Body Composition Assessment

**FIGURE 2:**
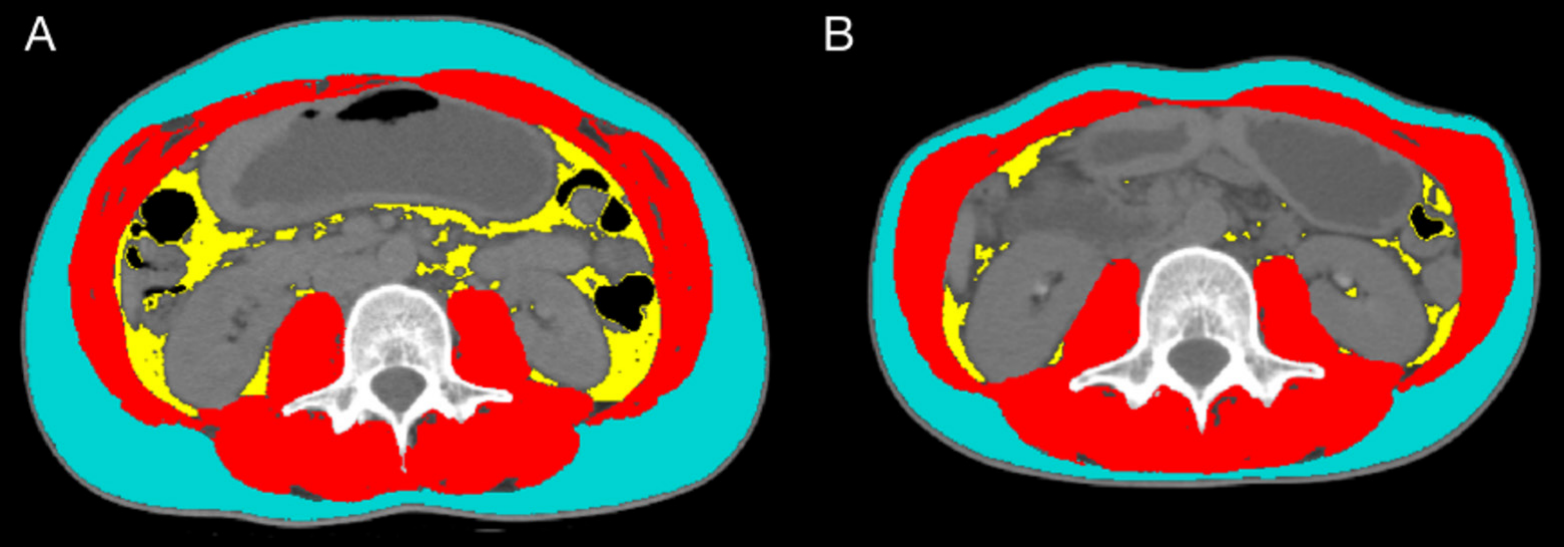
Cross‐section of pre‐NAC and post‐NAC CT‐scan images at the L3 region from the same patient. (A) Pre‐NAC. (B) Post‐NAC. Red: skeletal muscle; blue: subcutaneous adipose tissue; yellow: visceral adipose tissue. CT, computed tomography; NAC, neoadjuvant chemotherapy.

The pre‐NAC (baseline) CT scan is performed during the patient's initial consultation, which was closest to the first NAC circle, and the post‐NAC CT scan is conducted following the completion of NAC and prior to surgery. Skeletal muscle (SM), visceral adipose tissue (VAT) and subcutaneous adipose tissue (SAT) were analysed using axial CT images at the level of the third lumbar vertebra (L3) by SliceOmatic software (Version 5.0; Tomovision). Tissue Hounsfield unit (HU) thresholds were described previously: −29 to +150 HU for SM, −190 to −30 for SAT and −150 to −50 for VAT [10]. The mean tissue‐specific radiation attenuation (RA) of SM, VAT or SAT was recorded, and the area of SM, VAT and SAT was measured, and each value of the cross‐sectional areas (cm^2^) was normalized for height squared (m^2^) to calculate SM index (SMI), VAT index (VATI) or SAT index (SATI) (Figure 2), respectively. Sarcopenia and myosteatosis were evaluated based on CT at the L3 level by SMI and SMRA using predefined and frequently used cut‐off values [28]. Specifically, sarcopenia was defined in male patients as SMI < 43 cm^2^/m^2^ if BMI < 25 kg/m² and SMI < 53 cm^2^/m^2^ if BMI ≥ 25 kg/m^2^, and in female patients as SMI < 41 kg/m^2^ irrespective of BMI. Myosteatosis was defined as SMRA < 41 HU if BMI < 25 kg/m^2^ and SMRA < 33 HU if BMI ≥ 25 kg/m^2^ [29].

### 2.3 Outcomes and Follow‐Up

The follow‐up was ended in September 2019, and the median (range) follow‐up time was 61 (2–96) months in the SOX group and 60 (1–96) months in the FOLFOX group. After completion of the treatment, enrolled patients were followed up every 3–6 months from 1 to 2 years, 6 to 12 months from 3 to 5 years and then annually after 5 years. Patients' OS and progression‐free survival (PFS) were assessed. Whether the tumour recurred or progressed was determined by radiological findings or tissue biopsy, if it was feasible. Cause of death and sites of recurrence or progression were assessed and recorded by investigators.

### 2.4 Statistical Analysis

Continuous variables were presented as means ± standard deviations (SDs) or medians and interquartile ranges (IQRs) as appropriate for the data type. The Kolmogorov–Smirnov test was used to evaluate the normality of the data distribution. Normally distributed data were compared using Student's *t*‐tests, and non‐normally distributed continuous variables were compared using Mann–Whitney *U* tests or the Wilcoxon matched‐pairs signed rank test. Categorical variables are expressed as numbers (%) and were compared with the chi‐square test. A precision test was performed by analysing twice 30 images (15 pre‐NAC images repeated twice and 15 post‐NAC images repeated twice) according to a previously reported approach [30, 31]. Precision error was calculated by the percentage coefficient of variation (%CV) and by the root‐mean‐square (RMS) SD with the least significant change (LSC) for RMS %CV and for RMS SD. OS and PFS rates were calculated using the Kaplan–Meier method and compared using the log‐rank test. Kaplan–Meier survival analyses were conducted using the ‘survival’ and ‘survminer’ packages in R version 4.2.2 and the optimal cut‐off values were determined using the ‘maxstat’ package (https://CRAN.R‐project.org/package=maxstat), utilizing the methodology of maximum rank statistics. Statistical analyses were conducted using SPSS software, Version 26 (IBM, Armonk, NY USA). A *p* value < 0.05 was considered statistically significant.

## 3 Results

### 3.1 Baseline Characteristics of the Patients

A total of 583 patients from 12 Chinese hospitals were enrolled. After exclusion, 423 patients who proceeded to surgery were used for further analysis including 216 patients in the SOX group and 207 patients in the FOLFOX group (Figure 1). The baseline characteristics were shown in Table S1. The median age was 60 (IQR: 54–66) years, and 290 patients (68.6%) were male. In addition, the parameters of body composition were shown in Table 1. The baseline characteristics (including age, sex, ECOG performance status and surgical and pathological findings) were well balanced between the SOX group and the FOLFOX group.

**TABLE 1:** Patient characteristics of body composition parameters.

	Overall (*n* = 423)	SOX (*n* = 216)	FOLFOX (*n* = 207)	*p*
Pre‐sarcopenia (%)	132 (31.2)	64 (29.6)	68 (32.9)	0.542
Pre‐myosteatosis (%)	269 (63.6)	141 (65.3)	128 (61.8)	0.526
Pre‐SMI (mean [SD])	47.43 (8.03)	47.52 (8.11)	47.32 (7.97)	0.799
Pre‐VATI (median [IQR])	30.95 [16.67, 47.51]	31.69 [17.59, 47.74]	27.96 [15.40, 47.09]	0.219
Pre‐SATI (median [IQR])	34.84 [22.81, 47.64]	36.08 [24.50, 49.09]	33.73 [21.60, 46.92]	0.116
Pre‐SMRA (median [IQR])	37.72 [32.50, 41.71]	37.92 [32.42, 41.45]	37.60 [32.73, 42.22]	0.737
Pre‐VATRA (median [IQR])	−93.16 [−100.24, −85.28]	−94.39 [−101.43, −86.04]	−92.33 [−99.04, −84.50]	0.102
Pre‐SATRA (median [IQR])	−94.99 [−101.10, −87.53]	−95.83 [−101.60, −88.62]	−94.68 [−100.80, −86.36]	0.164
Post‐sarcopenia (%)	158 (37.4)	91 (42.1)	67 (32.4)	0.048
Post‐myosteatosis (%)	278 (65.7)	143 (66.2)	135 (65.2)	0.911
Post‐SMI (mean [SD])	46.62 (8.09)	46.42 (8.27)	46.82 (7.92)	0.608
Post‐VATI (median [IQR])	28.19 [17.11, 44.11]	28.46 [17.62, 43.90]	27.34 [16.30, 44.65]	0.651
Post‐SATI (median [IQR])	32.41 [22.48, 45.27]	33.42 [23.09, 47.12]	31.68 [22.11, 43.30]	0.302
Post‐SMRA (median [IQR])	36.41 [31.44, 40.97]	36.38 [30.79, 40.65]	36.46 [31.96, 41.20]	0.661
Post‐VATRA (median [IQR])	−88.98 [−95.21, −83.39]	−88.91 [−95.08, −82.82]	−89.18 [−95.35, −84.22]	0.614
Post‐SATRA (median [IQR])	−92.48 [−97.71, −85.03]	−92.92 [−98.19, −84.34]	−92.34 [−97.54, −85.46]	0.909

Abbreviations: FOLFOX, fluorouracil, leucovorin and oxaliplatin; IQR, interquartile range; SATI, subcutaneous adipose tissue index; SATRA, subcutaneous adipose tissue radiation attenuation; SD, standard deviation; SMI, skeletal muscle index; SMRA, skeletal muscle radiation attenuation; SOX, S‐1 and oxaliplatin; VATI, visceral adipose tissue index; VATRA, visceral adipose tissue radiation attenuation.

### 3.2 The Clinical Characteristics in Patients With Sarcopenia or Myosteatosis

According to predefined criteria, 132 patients (31.2%) were diagnosed with sarcopenia and 269 patients (63.6%) were diagnosed with myosteatosis. The characteristics of the patients according to sarcopenia or myosteatosis were shown in Table 2. Female patients were more prone to sarcopenia (*p* < 0.001) or myosteatosis (*p* < 0.001). Patients with sarcopenia and myosteatosis were older than those without (*p* = 0.048; *p* < 0.001). Sarcopenic patients had a lower BMI than non‐sarcopenic patients (*p* < 0.001). Either sarcopenia or myosteatosis affected performance status and was associated with the elevated ECOG scores (*p* = 0.025; *p* = 0.019). There was no significant difference in surgical and pathological characteristics between sarcopenia patients and non‐sarcopenia patients, and these characteristics were also balanced between myosteatosis patients and non‐myosteatosis patients.

**TABLE 2:** Patient characteristics based on sarcopenia or myosteatosis group.

	Overall (*n* = 423)	Non‐sarcopenia (*n* = 291)	Sarcopenia (*n* = 132)	*p*	Non‐myosteatosis (*n* = 154)	Myosteatosis (*n* = 269)	*p*
Gender (%)				< 0.001			< 0.001
Male	290 (68.6)	232 (79.7)	58 (43.9)		130 (84.4)	160 (59.5)	
Female	133 (31.4)	59 (20.3)	74 (56.1)		24 (15.6)	109 (40.5)	
Age (median [IQR])	60.00 [54.00, 66.00]	60.00 [54.00, 66.00]	62.50 [53.50, 68.00]	0.048	56.00 [48.25, 63.00]	63.00 [57.00, 68.00]	< 0.001
BMI (median [IQR])	22.66 [20.70, 24.57]	23.03 [21.26, 24.86]	21.91 [19.83, 24.03]	< 0.001	22.50 [20.20, 25.95]	22.76 [20.96, 24.22]	0.803
ECOG (%)				0.025			0.019
0	232 (54.8)	168 (57.7)	64 (48.5)		97 (63.0)	135 (50.2)	
1	182 (43.0)	120 (41.2)	62 (47.0)		56 (36.4)	126 (46.8)	
2	9 (2.1)	3 (1.0)	6 (4.5)		1 (0.6)	8 (3.0)	
Location (%)				0.689			0.736
Upper	57 (13.5)	43 (14.8)	14 (10.6)		18 (11.7)	39 (14.5)	
Middle	84 (19.9)	57 (19.6)	27 (20.5)		34 (22.1)	50 (18.6)	
Lower	258 (61.0)	174 (59.8)	84 (63.6)		94 (61.0)	164 (61.0)	
Diffuse type	24 (5.7)	17 (5.8)	7 (5.3)		8 (5.2)	16 (5.9)	
Type of gastrectomy (%)				0.160			0.450
Distal	252 (59.6)	167 (57.4)	85 (64.4)		94 (61.0)	158 (58.7)	
Total	166 (39.2)	119 (40.9)	47 (35.6)		57 (37.0)	109 (40.5)	
Unknown	5 (1.2)	5 (1.7)	0 (0.0)		3 (1.9)	2 (0.7)	
Combined resection (%)	15 (3.6)	10 (3.4)	5 (3.8)	0.395	4 (2.6)	11 (4.1)	0.624
Lymphadenectomy (%)				0.106			0.333
D1	3 (0.7)	1 (0.3)	2 (1.5)		0 (0.0)	3 (1.1)	
D2	414 (97.9)	284 (97.6)	130 (98.5)		151 (98.1)	263 (97.8)	
Unknown	6 (1.4)	6 (2.1)	0 (0.0)		3 (1.9)	3 (1.1)	
Type of anastomosis (%)				0.182			0.455
Billroth‐I	5 (1.2)	2 (0.7)	3 (2.3)		1 (0.6)	4 (1.5)	
Billroth‐II	244 (57.7)	164 (56.4)	80 (60.6)		93 (60.4)	151 (56.1)	
Roux‐en‐Y	169 (40.0)	120 (41.2)	49 (37.1)		57 (37.0)	112 (41.6)	
Unknown	5 (1.2)	5 (1.7)	0 (0.0)		3 (1.9)	2 (0.7)	
Tumour residual (%)				0.160			0.163
R0	403 (95.3)	273 (93.8)	130 (98.5)		149 (96.8)	254 (94.4)	
R1	4 (0.9)	4 (1.4)	0 (0.0)		0 (0.0)	4 (1.5)	
R2	11 (2.6)	9 (3.1)	2 (1.5)		2 (1.3)	9 (3.3)	
Unknown	5 (1.2)	5 (1.7)	0 (0.0)		3 (1.9)	2 (0.7)	
TNM (%)				0.374			0.806
pCR	10 (2.4)	8 (2.7)	2 (1.5)		5 (3.2)	5 (1.9)	
I	86 (20.3)	64 (22.0)	22 (16.7)		30 (19.5)	56 (20.8)	
II	104 (24.6)	65 (22.3)	39 (29.5)		37 (24.0)	67 (24.9)	
III	196 (46.3)	133 (45.7)	63 (47.7)		69 (44.8)	127 (47.2)	
IV	13 (3.1)	9 (3.1)	4 (3.0)		6 (3.9)	7 (2.6)	
Not evaluable	9 (2.1)	7 (2.4)	2 (1.5)		4 (2.6)	5 (1.9)	
Unknown	5 (1.2)	5 (1.7)	0 (0.0)		3 (1.9)	2 (0.7)	

Abbreviations: IQR, interquartile range; pCR, pathological complete response.

Furthermore, we evaluated the prognostic role of sarcopenia or myosteatosis based on Kaplan–Meier survival analyses. The 1‐, 3‐ and 5‐year PFS rates were 85.6%, 70.5% and 67.4% in the sarcopenia group, respectively, which showed no significant difference with 85.9%, 72.7% and 70.6% in the non‐sarcopenia group, respectively (*p* = 0.563, Figure S1A). There was also no significant difference in OS between these two groups (*p* = 0.363; Figure S1B). Likewise, there was no significant discrepancy observed in either PFS (*p* = 0.985; Figure S1C) or OS (*p* = 0.789; Figure S1D) between myosteatosis patients and non‐myosteatosis patients.

In addition, we reassessed the body status of patients after the NAC based on the post‐NAC CT images. After the NAC, more patients suffered sarcopenia or myosteatosis. A total of 158 patients (37.4%) were diagnosed with sarcopenia, and 278 patients (65.7%) were diagnosed with myosteatosis. Patients with sarcopenia exhibited a tendency to have reduced PFS (*p* = 0.068; Figure S2A) and markedly reduced OS (*p* = 0.031;l Figure S2B) compared to those without sarcopenia. However, there was still no significant difference observed in either PFS (*p* = 0.571, Figure S2C) or OS (*p* = 0.798; Figure S2D) between patients with myosteatosis and those without myosteatosis.

### 3.3 Dynamic Change of Body Composition During NAC

**FIGURE 3:**
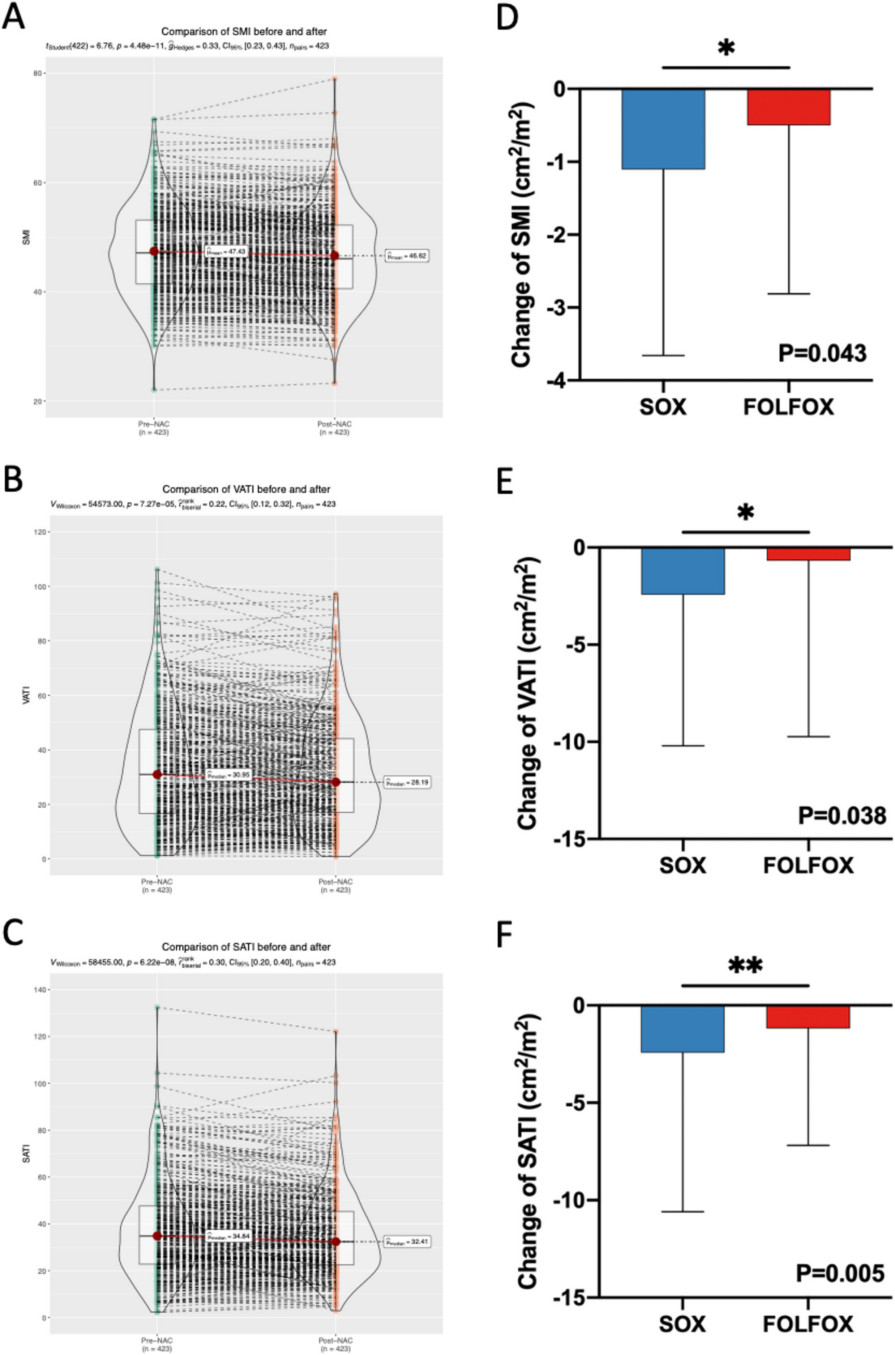
Dynamic change of body composition parameters before and after the NAC. Comparison of SMI (A), SATI (B) and VATI (C) before and after the NAC. Change of SMI (D), SATI (E) and VATI (F) in SOX and FOLFOX group. *p < 0.05, **p < 0.01. FOLFOX, fluorouracil, leucovorin and oxaliplatin; NAC, neoadjuvant chemotherapy; SATI, subcutaneous adipose tissue index; SMI, skeletal muscle index; SOX, S‐1 and oxaliplatin; VATI, visceral adipose tissue index.

To investigate the impact of dynamic changes in body composition on patient outcomes, we measured and compared the pre‐NAC and post‐NAC body composition parameters. The median interval between these two images is 55 days (IQR, 48–65). The precision error and the LSC of SM or SAT measurements are presented in Table S2. The LSC %CV for SM area, SAT area and VAT area was 2.01%, 2.06% and 3.43%, respectively. After the NAC, most patients showed reduced SMI, SATI and VATI (Figure 3A–C, Figures S3A–C and S4), and the change showed no association with NAC cycle number (Figure S3D–F). The reduction of SMI, SATI or VATI in the SOX group was more than that in the FOLFOX group (P_SMI_ = 0.043, P_VATI_ = 0.038, P_SATI_ = 0.005, respectively) (Figure 3D–F). Additionally, the proportion of sarcopenia patients after the NAC was significantly higher in the SOX group than that of the FOLFOX group (*p* = 0.048), although the baseline sarcopenic proportions were comparable in the two groups (*p* = 0.542) (Table 1).

### 3.4 Prognostic Role of Adipose or Muscle Loss During NAC

**FIGURE 4:**
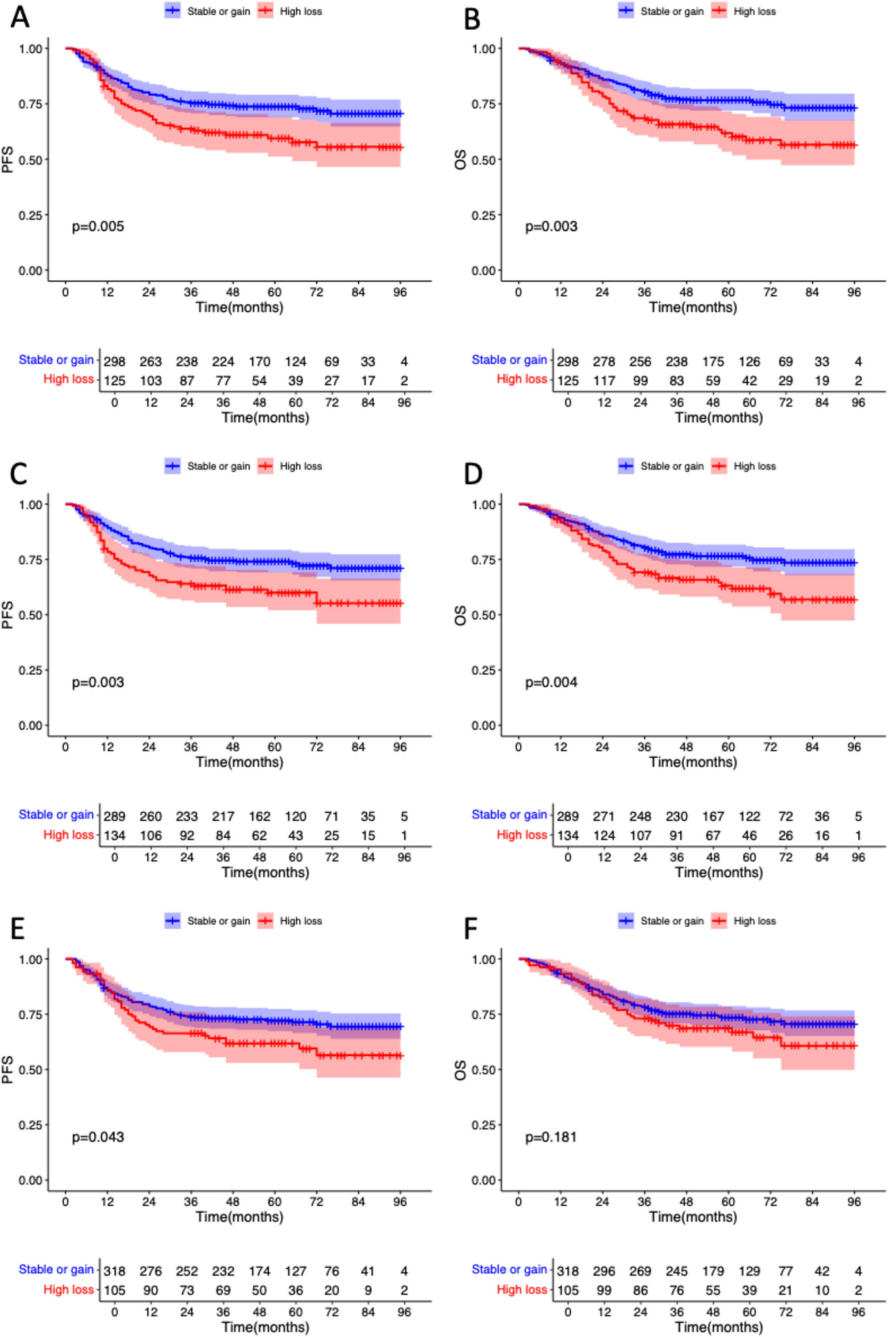
Kaplan–Meier curves for high loss group and stable or gain group. (A) PFS according to SATI loss. (B) OS according to SATI loss. (C) PFS according to VATI loss. (D) OS according to VATI loss. (E) PFS according to SMI loss. (F) OS according to SMI loss. OS, overall survival; PFS, progression‐free survival; SATI, subcutaneous adipose tissue index; SMI, skeletal muscle index; VATI, visceral adipose tissue index.

To assess the prognostic role of body composition dynamic change, we divided patients into two groups (high loss: *n* = 125; stable or gain: *n* = 298) according to the SATI loss percentage, and the optimal cut‐off value was 12.5% determined using the ‘maxstat’ package. Patients with SATI loss > 12.5% had significantly worse PFS (*p* = 0.005; Figure 4A) and OS (*p* = 0.003; Figure 4B) than those without. Using the same method, the optimal cut‐off value of VATI loss was 15%, and patients with VATI loss > 15% (*n* = 134, 31.7%) had significantly worse PFS (*p* = 0.003; Figure 4C) and OS (*p* = 0.004; Figure 4D) than the stable or gain group. According to the SMI loss percentage, we also divided patients into two groups (high loss: *n* = 105; stable or gain: *n* = 318), and the optimal cut‐off value was 4.7%. Patients with SMI loss > 4.7% had significantly worse PFS (*p* = 0.043; Figure 4E) and showed a tendency of reduced OS (*p* = 0.181; Figure 4F) than those without.

To sum up, both high loss of adipose tissue and skeletal muscle during NAC were associated with adverse survival outcomes, and high loss of adipose tissue could better differentiate patients with poor prognosis.

### 3.5 Subgroup Analysis of Chemotherapy‐Associated Toxic Effects

Furthermore, to assess the chemotherapy‐associated toxic effects of these two regimens on patients with different baseline body composition status, we divided patients into two groups according to sarcopenia or myosteatosis and conducted subgroup analysis (Table S3). In non‐sarcopenic patients, we observed significantly more instances of Grade 3 or 4 neutrocytopenia in the FOLFOX group (*p* = 0.019). In patients with myosteatosis, the incidence of thrombocytopenia in the SOX group was higher than that of the FOLFOX group (*p* < 0.001). In patients without myosteatosis, there were significantly more instances of Grade 3 or 4 anaemia in the SOX group (*p* = 0.043).

## 4 Discussion

Based on this RCT trial, we demonstrated that patients with GC often have significant changes in body composition during NAC, including a high‐level loss of adipose tissue or skeletal muscle, which was associated with significantly poor prognosis. In addition, the baseline body composition status was also related to chemotherapy‐associated toxic effects. To our knowledge, it is the first trial that investigated the prognostic role of body composition parameters in GC patients who received NAC of SOX or FOLFOX regimen systematically.

Cachexia is highly prevalent in patients with gastric cancer. Taking the extratumoral effects of cancer into account (host response to cancer) may be valuable to tumour stage for risk stratification and identification of patients with potential poor prognosis. A recent multicentre cohort study revealed that rates of disease progression during NAC, leading to inoperability, were higher in patients with a low cachexia index (28% vs. 12%, *p* < 0.001), and cachexia was associated with worsened post‐operative mortality and decreased OS in patients with locally advanced oesophageal or gastric cancer [32]. According to the definition of cachexia, muscle loss is a feature of cachexia. Previous studies have focused on muscle, and a significant number of patients with GC become sarcopenic during NAC [33]. A recent study revealed that both of pre‐NAC sarcopenia and post‐NAC sarcopenia were associated with the high post‐operative morbidity rates and post‐NAC sarcopenia was an independent risk factor for post‐operative morbidity (HR: 9.550; 95% CI 2.818–32.369; *p* < 0.001) [34]. In addition, sarcopenia is also associated with early termination of NAC, a significantly extended post‐operative stay, hospital stay and severe post‐operative complications [35, 36]. SMI loss > 1.2 cm^2^/m^2^ during NAC independently predicted poor OS (HR: 1.677, 95% CI 1.040–2.704, *p* = 0.034) and recurrence‐free survival (HR: 1.924, 95% CI 1.165–3.175, *p* = 0.011) [37]. A previous study demonstrated that muscle loss > 6% was associated with reduced survival [25]. Muscle loss ≥ 5% during NAC was associated with a higher Mandard tumour regression grade, which is a histological score of response after NAC, and the higher is the grade, the worse is the response [26, 38]. The adverse host response may be interpreted by the adverse tumour biology. In addition, muscle mass may be involved in the drug pharmacokinetics and the synthesis of several acute‐phase proteins, and a better pathological response may contribute to a reduction in dysphagia and then a better nutritional intake [26, 39]. The cut‐off value of SMI loss is 4.7% in our study, and it was associated with worse PFS (*p* = 0.043) and only showed a tendency of reduced OS (*p* = 0.181). Muscle loss seems not to be the most important factor in differentiating patients with poor prognosis in our trial. The reason is related to the fact that adipose tissue tends to be consumed first, followed by muscle loss.

Adipose tissue is also a significant evaluation factor of body composition and was suggested as having a prognostic role in GC patients. Recently, a retrospective cohort study (*n* = 341) revealed that a low VFI (cut‐off value: 41.28 cm^2^/m^2^) was an independent risk factor associated with the poor OS (HR: 1.59; 95% CI 1.03–2.45; *p* = 0.038) in patients with GC (Stages I–III) who underwent gastrectomy, and the CT time was within 1 month prior to surgery [40]. A retrospective study (*n* = 157) found that patients with marked VAT loss (≥ 35.7%) during NAC or neoadjuvant chemoradiotherapy had significantly shorter OS (*p* = 0.028) and marked SAT loss (≥ 30.1%) was also associated with poorer OS (*p* = 0.033) and DFS (*p* = 0.003) [41]. The proportion of the high SAT loss group and high VAT group was 12.1% (*n* = 19) and 10.2% (*n* = 16), respectively. In our study, the cut‐off value was 12.5% for high SAT loss and 15% for high VAT loss, and the percentage of the high SAT loss group and high VAT group was 29.6% (*n* = 125) and 31.7% (*n* = 134), respectively. We extended the criteria and figured out more GC patients with potential poor prognosis. In clinical practice, we recommend assessing and monitoring body composition parameters and their dynamic changes based on pre‐NAC CT images and the post‐NAC CT images. The main purpose of pre‐NAC CT is to assist in the diagnosis and clinical staging of GC, and the post‐NAC CT can be used to assess the efficacy of NAC to some degree. The assessment of body composition does not necessitate additional CT imaging; instead, it facilitates the extraction of prognostic information from the existing data. For patients who suffered muscle and fat loss exceeding our cut‐off value, individualized therapy, dietary guidance, exercise programmes and early nutritional intervention should be considered to mitigate these effects.

NAC followed by radical surgery is recommended as the standard treatment for locally advanced GC [42, 43, 44]. SOX and FOLFOX regimens were found to be effective and manageable with a favourable safety profile in patients with unresectable GC in Asia [5, 45, 46]. As we reported previously, SOX was non‐inferior to FOLFOX for patients with locally advanced GC, and the 3‐year OS rate was 75.2% for the SOX group and 67.8% for the FOLFOX group [27]. SOX is the Grade I recommendation for non‐oesophagogastric junction locally advanced GC according to the Chinese Society of Clinical Oncology (CSCO) clinical guideline [44]. However, we revealed that the SOX group showed a remarkable reduction of skeletal muscle and adipose tissue after NAC, and the proportion of sarcopenia patients after the NAC was significantly higher in the SOX group than that of the FOLFOX group. The reason is related to the fact that S‐1 was given continuously twice daily for 2 weeks. These results remind us that body composition status should be taken into account when choosing the NAC regimen in clinical practice. In addition, the chemotherapy‐associated toxic effects differed among patients with different baseline body composition statuses. Prevention and in‐time intervention of complications should be adopted in specific subgroups.

Admittedly, potential limitations of our study must also be considered. Body condition requires multidimensional evaluation such as grip strength, physical performance and nutritional assessment questionnaire, which were unavailable in our study. Furthermore, we investigated the prognostic role of dynamic change of body composition during NAC, although surgery may have a greater impact on body composition and physical function. Hence, further longitudinal studies are warranted to clarify the association between perioperative change of body composition and outcomes.

## 5 Conclusion

In conclusion, we found that high loss of adipose tissue during NAC played a prognostic role in patients with locally advanced GC, which was better than the prognostic role of muscle loss. The SOX regimen caused more muscle and fat consumption than the FOLFOX regimen. Monitoring body composition change during NAC is helpful to predict prognosis and effectively guide individual nutrition intervention. Body composition status should be taken into account when choosing the NAC regimen in clinical practice.

## Ethics Statement

The study was done according to the Declaration of Helsinki and Good Clinical Practice Guidelines as defined by the International Conference on Harmonization. All enrolled patients provided written informed consent. The trial is registered at ClinicalTrials.gov (NCT01364376). The study protocol was approved by the ethics committees of participating institutions based on the ethical approval obtained from the ethics committee of the lead centre, the First Affiliated Hospital, Zhejiang University School of Medicine (approval number: ZDYYLS2024Y, No. 1105‐K).

## Conflicts of Interest

The authors declare no conflicts of interest.

## Supporting information


**Figure S1:** Kaplan–Meier curves for survival in patients with sarcopenia or myosteatosis based on pre‐NAC CT.
**Figure S2:** Kaplan–Meier curves for survival in patients with sarcopenia or myosteatosis based on post‐NAC CT.
**Figure S3:** Change percentage of SMI, SATI and VATI during NAC and the association with number of NAC cycles.
**Figure S4:** Change of SMI, SATI and VATI during NAC.
**Table S1:** Baseline characteristics.
**Table S2:** Precision error and least significant change of the cross‐sectional area and radiation attenuation for muscle and adipose tissue.
**Table S3:** Grade 3/4 chemotherapy‐associated toxic effects of SOX or FOLFOX on patients with different baseline body composition status.

**Data S1:** Supporting Information.
