# Supplementary material for: High Loss of Adipose Tissue During Neoadjuvant Chemotherapy Predicts Poor Prognosis in Patients With Gastric Cancer
Source: J Cachexia Sarcopenia Muscle. 2025 Oct 30;16(6):e70107. doi: 10.1002/jcsm.70107 (PMC12575444; doi:10.1002/jcsm.70107)
Supplement: Supplementary file 1 — Figure S1: Kaplan–Meier curves for survival in patients with sarcopenia or myosteatosis based on pre‐NAC CT. Figure S2: Kaplan–Meier curves for survival in patients with sarcopenia or myosteatosis based on post‐NAC CT. Figure S3: Change percentage of SMI, SATI and VATI during NAC and the association with number of NAC cycles. Figure S4: Change of SMI, SATI and VATI during NAC. Table S1: Baseline characteristics. Table S2: Precision error and least significant change of the cross‐sectional area and radiation attenuation for muscle and adipose tissue. Table S3: Grade 3/4 chemotherapy‐associated toxic effects of SOX or FOLFOX on patients with different baseline body composition status. [file JCSM-16-e70107-s001.docx]

**Supplemental material**

**Figure S1. Kaplan-Meier curves for survival in patients with sarcopenia or myosteatosis based on pre-NAC CT.**

**Figure S2. Kaplan-Meier curves for survival in patients with sarcopenia or myosteatosis based on post-NAC CT.**

**Figure S3. Change percentage of SMI, SATI and VATI during NAC and the association with number of NAC cycles.**

**Figure S4. Change of SMI, SATI and VATI during NAC.**

**Table S1. Baseline characteristics.**

**Table S2. Precision error and least significant change of the cross-sectional area and radiation attenuation for muscle and adipose tissue.**

**Table S3. Grade 3/4 chemotherapy-associated toxic effects of SOX or FOLFOX on patients with different baseline body composition status.**


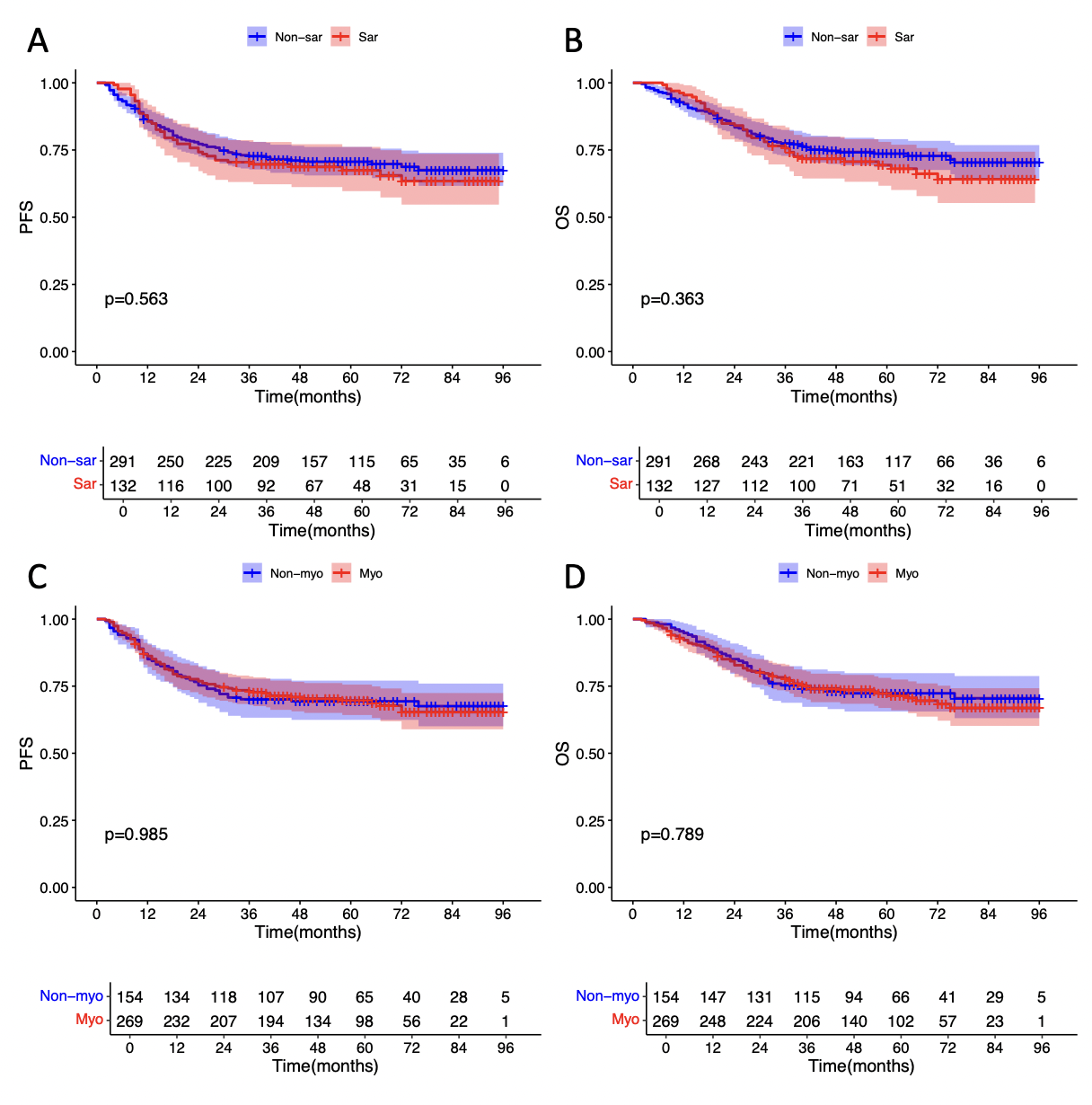


**Figure S1. Kaplan-Meier curves for survival in patients with sarcopenia or myosteatosis based on pre-NAC CT. (A) PFS in patients with or without sarcopenia; (B) OS in patients with or without sarcopenia; (C) PFS in patients with or without myosteatosis; (D) OS in patients with or without myosteatosis.** Abbreviations: Myo, myosteatosis; OS, overall survival; PFS, progression-free survival; Sar, sarcopenia.

**
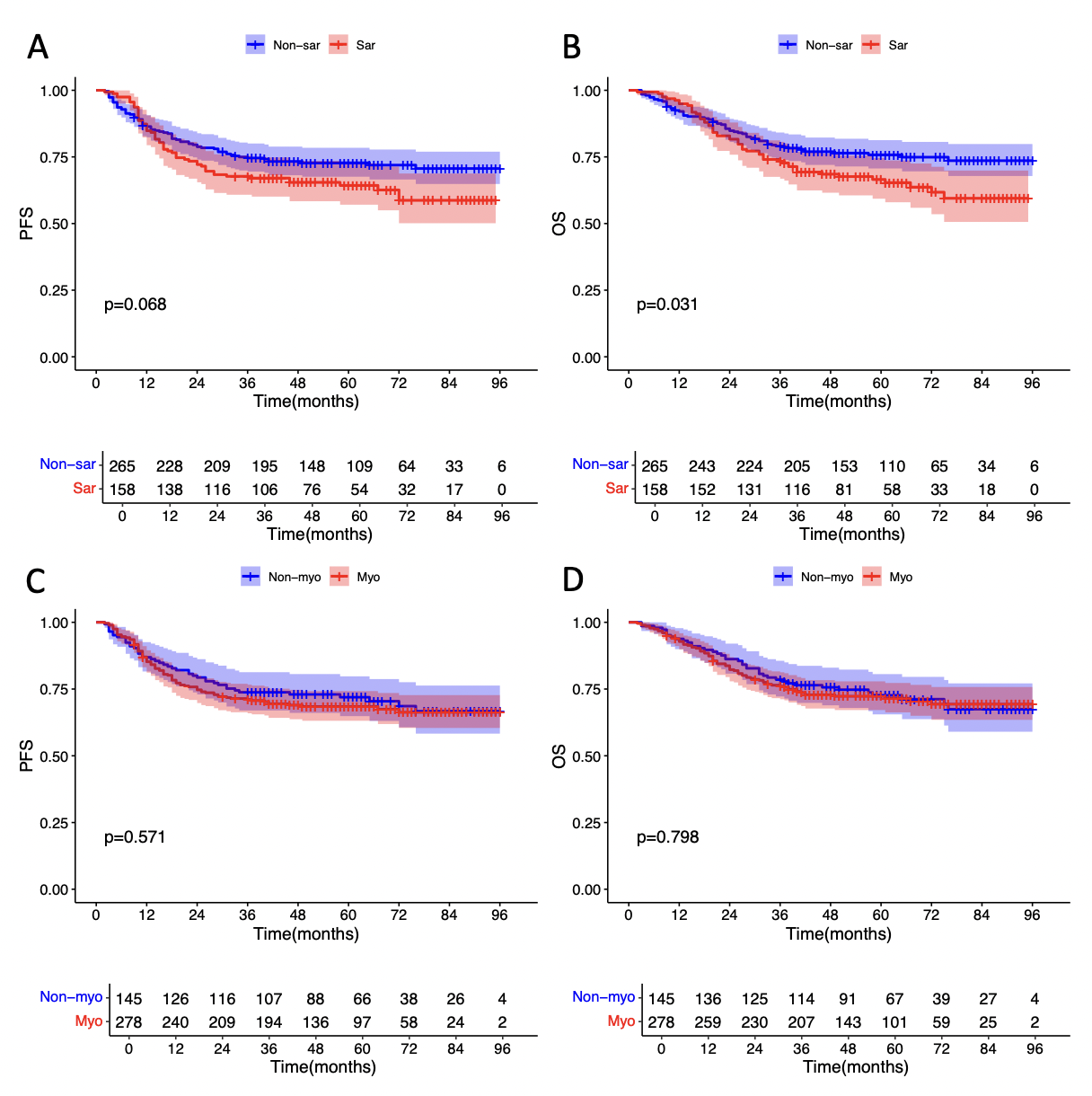
**

**Figure S2. Kaplan-Meier curves for survival in patients with sarcopenia or myosteatosis based on post-NAC CT. (A) PFS in patients with or without sarcopenia; (B) OS in patients with or without sarcopenia; (C) PFS in patients with or without myosteatosis; (D) OS in patients with or without myosteatosis.** Abbreviations: Myo, myosteatosis; OS, overall survival; PFS, progression-free survival; Sar, sarcopenia.

**
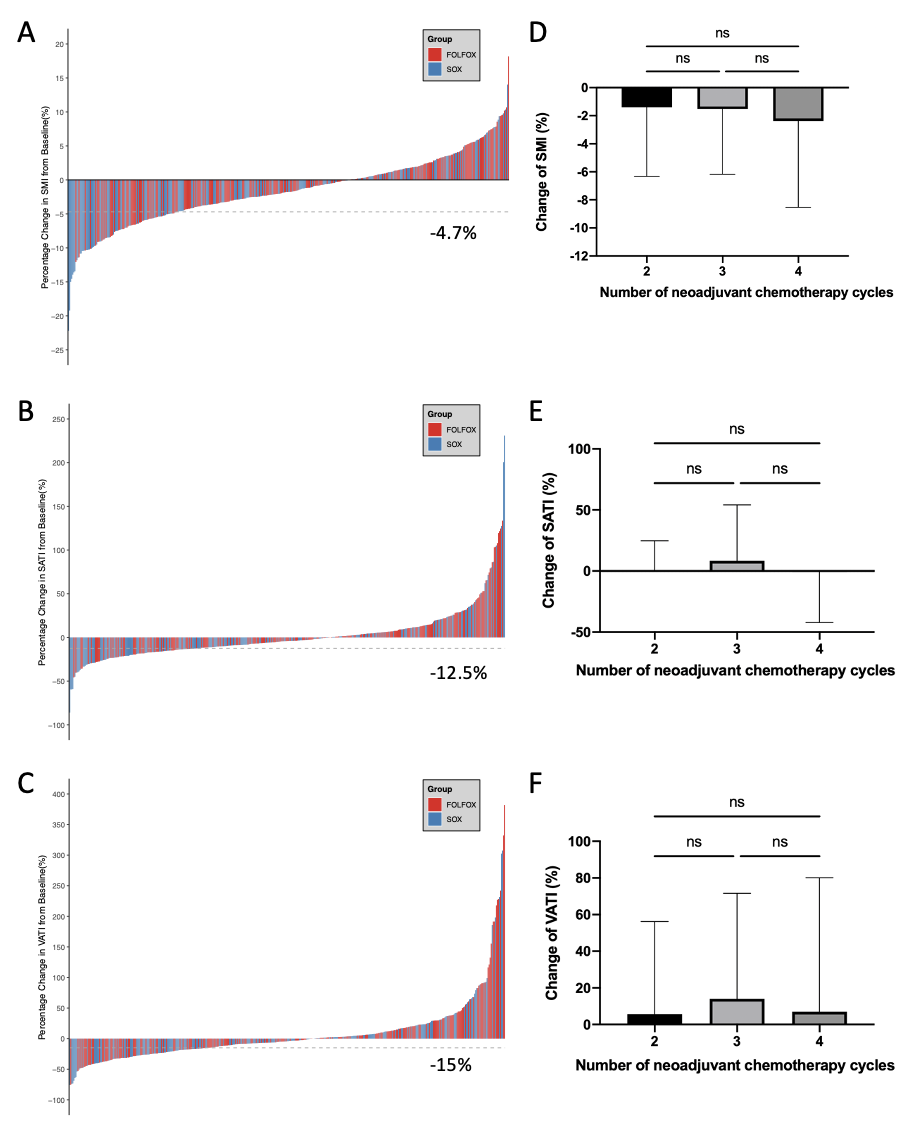
**

**Figure S3. Change percentage of SMI, SATI and VATI during NAC and the association with number of NAC cycles. (A) Percentage change in SMI; (B) Percentage change in SATI; (C) Percentage change in VATI; The association between number of NAC cycles and change of SMI (D), SATI(E) and VATI (F).** Abbreviations: FOLFOX, fluorouracil, leucovorin and oxaliplatin; NAC, neoadjuvant chemotherapy; ns, no significance; SATI, subcutaneous adipose tissue index; SMI, skeletal muscle index; SOX, S-1 and oxaliplatin; VATI, visceral adipose tissue index.


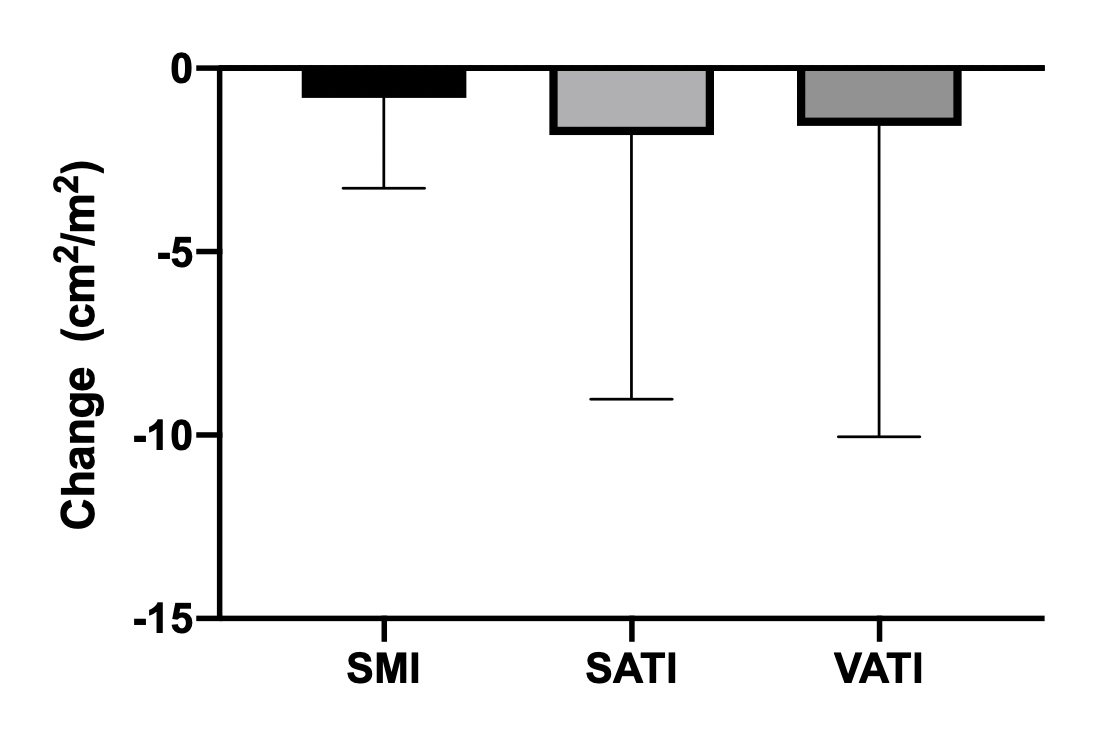


**Figure S4. Change of SMI, SATI and VATI during NAC.** Abbreviations: NAC, neoadjuvant chemotherapy; SATI, subcutaneous adipose tissue index; SMI, skeletal muscle index; VATI, visceral adipose tissue index.

| **Table S1. Baseline characteristics.** | | | | |
| --- | --- | --- | --- | --- |
|  | Overall (n=423) | SOX (n=216) | FOLFOX (n=207) | P value |
| Gender (%) |  |  |  | 0.112 |
| Male | 290 (68.6) | 140 (64.8) | 150 (72.5) |  |
| Female | 133 (31.4) | 76 (35.2) | 57 (27.5) |  |
| age (median [IQR]) | 60.00 [54.00, 66.00] | 60.50 [53.00, 67.00] | 60.00 [54.00, 66.00] | 0.868 |
| BMI (median [IQR]) | 22.66 [20.70, 24.57] | 22.62 [20.56, 24.83] | 22.72 [20.92, 24.38] | 0.770 |
| ECOG (%) |  |  |  | 0.515 |
| 0 | 232 (54.8) | 121 (56.0) | 111 (53.6) |  |
| 1 | 182 (43.0) | 89 (41.2) | 93 (44.9) |  |
| 2 | 9 (2.1) | 6 (2.8) | 3 (1.4) |  |
| Primary tumor location (%) |  |  |  | 0.243 |
| Upper | 57 (13.5) | 36 (16.7) | 21 (10.1) |  |
| Middle | 84 (19.9) | 42 (19.4) | 42 (20.3) |  |
| Lower | 258 (61.0) | 125 (57.9) | 133 (64.3) |  |
| Diffuse type | 24 (5.7) | 13 (6.0) | 11 (5.3) |  |
| Type of gastrectomy (%) |  |  |  | 0.063 |
| Distal | 252 (59.6) | 118 (54.6) | 134 (64.7) |  |
| Total | 166 (39.2) | 94 (43.5) | 72 (34.8) |  |
| Unknown | 5 (1.2) | 4 (1.9) | 1 (0.5) |  |
| Combined resection (%) | 15 (3.6) | 8 (3.8) | 7 (3.4) | 0.999 |
| Lymphadenectomy (%) |  |  |  | 0.235 |
| D1 | 3 (0.7) | 1 (0.5) | 2 (1.0) |  |
| D2 | 414 (97.9) | 210 (97.2) | 204 (98.6) |  |
| Unknown | 6 (1.4) | 5 (2.3) | 1 (0.5) |  |
| Type of anastomosis (%) |  |  |  | 0.156 |
| Billroth-I | 5 (1.2) | 2 (0.9) | 3 (1.4) |  |
| Billroth-II | 244 (57.7) | 115 (53.2) | 129 (62.3) |  |
| Roux-en-Y | 169 (40.0) | 95 (44.0) | 74 (35.7) |  |
| Unknown | 5 (1.2) | 4 (1.9) | 1 (0.5) |  |
| Tumor residual (%) |  |  |  | 0.623 |
| R0 | 403 (95.3) | 204 (94.4) | 199 (96.1) |  |
| R1 | 4 (0.9) | 2 (0.9) | 2 (1.0) |  |
| R2 | 11 (2.6) | 6 (2.8) | 5 (2.4) |  |
| Unknown | 5 (1.2) | 4 (1.9) | 1 (0.5) |  |
| TNM stage (%) |  |  |  | 0.109 |
| pCR | 10 (2.4) | 8 (3.7) | 2 (1.0) |  |
| Ⅰ | 86 (20.3) | 44 (20.4) | 42 (20.3) |  |
| Ⅱ | 104 (24.6) | 57 (26.4) | 47 (22.7) |  |
| Ⅲ | 196 (46.3) | 90 (41.7) | 106 (51.2) |  |
| Ⅳ | 13 (3.1) | 6 (2.8) | 7 (3.4) |  |
| Not evaluable | 9 (2.1) | 7 (3.2) | 2 (1.0) |  |
| Unknown | 5 (1.2) | 4 (1.9) | 1 (0.5) |  |

Abbreviations: FOLFOX, fluorouracil, leucovorin and oxaliplatin; IQR, interquartile range; pCR, pathological complete response; SOX, S-1 and oxaliplatin.

| **Table S2. Precision error and least significant change of the cross-sectional area and radiation attenuation for muscle and adipose tissue.** | | | | |
| --- | --- | --- | --- | --- |
|  | Precision | | LSC | |
|  | SD-RMS | %CV-RMS | LSC RMS SD | LSC % CV |
| SM area | 0.96 | 0.73 | 2.67 | 2.01 |
| SMRA | 0.41 | 0.66 | 1.13 | 1.83 |
| SAT area | 0.32 | 0.74 | 0.90 | 2.06 |
| SATRA | 0.18 | 0.21 | 0.50 | 0.58 |
| VAT area | 0.92 | 1.24 | 2.56 | 3.43 |
| VATRA | 0.47 | 0.54 | 1.30 | 1.51 |
| Abbreviations: CV, coefficient of variation; LSC, least significant change; RMS, root-mean-square; SAT, subcutaneous adipose tissue; SATRA, subcutaneous adipose tissue radiation attenuation; SD, standard deviation; SM, skeletal muscle; SMRA, skeletal muscle radiation attenuation; VAT, visceral adipose tissue; VATRA, visceral adipose tissue radiation attenuation. | | | | |

| **Table S3. Grade 3/4 chemotherapy-associated toxic effects of SOX or FOLFOX on patients with different baseline body composition status.** | | | | | | | | | | | | |
| --- | --- | --- | --- | --- | --- | --- | --- | --- | --- | --- | --- | --- |
|  | Sarcopenia | | | Non-sarcopenia | | | Myosteatosis | | | Non-myosteatosis | | |
|  | SOX  (n=64） | FOLFOX  (n=68) | P value | SOX  (n=152) | FOLFOX  (n=139) | P value | SOX  (n=141) | FOLFOX (n=129) | P value | SOX (n=75) | FOLFOX (n=78) | P value |
| Hematologic |  |  |  |  |  |  |  |  |  |  |  |  |
| Leukocytopenia | 5 | 2 | 0.212 | 8 | 4 | 0.307 | 10 | 6 | 0.396 | 3 | 0 | 0.074 |
| Neutrocytopenia | 18 | 20 | 0.870 | 28 | 42 | **0.019** | 32 | 37 | 0.260 | 14 | 25 | 0.058 |
| Lymphopenia | 4 | 4 | 0.930 | 5 | 2 | 0.303 | 4 | 3 | 0.792 | 5 | 3 | 0.433 |
| Thrombocytopenia | 15 | 3 | **0.001** | 14 | 2 | **0.004** | 23 | 3 | **<0.001** | 6 | 2 | 0.131 |
| Anemia | 11 | 9 | 0.527 | 15 | 7 | 0.119 | 18 | 14 | 0.627 | 8 | 2 | **0.043** |
| ALT | 0 | 2 | 0.167 | 2 | 4 | 0.349 | 1 | 4 | 0.145 | 1 | 2 | 0.583 |
| AST | 0 | 1 | 0.306 | 0 | 0 | / | 0 | 1 | 0.295 | 0 | 0 | / |
| Creatinine | 0 | 0 | / | 0 | 0 | / | 0 | 0 | / | 0 | 0 | / |
| Non-hematologic |  |  |  |  |  |  |  |  |  |  |  |  |
| Vomiting | 5 | 12 | 0.092 | 13 | 13 | 0.811 | 9 | 16 | 0.088 | 9 | 9 | 0.929 |
| Nausea | 7 | 5 | 0.474 | 14 | 9 | 0.388 | 12 | 10 | 0.820 | 9 | 4 | 0.128 |
| Fatigue | 0 | 1 | 0.330 | 3 | 1 | 0.359 | 2 | 1 | 0.614 | 1 | 1 | 0.978 |
| Fever | 1 | 1 | 0.966 | 0 | 1 | 0.295 | 1 | 1 | 0.950 | 0 | 1 | 0.325 |
| Hand-foot syndrome | 0 | 0 | / | 0 | 2 | 0.138 | 0 | 2 | 0.148 | 0 | 0 | / |
| Anorexia | 0 | 1 | 0.330 | 1 | 0 | 0.338 | 1 | 1 | 0.976 | 0 | 0 | / |
| Constipation | 0 | 0 | / | 1 | 0 | 0.338 | 0 | 0 | / | 1 | 0 | 0.306 |
| Diarrhea | 1 | 0 | 0.301 | 4 | 2 | 0.474 | 3 | 1 | 0.358 | 2 | 1 | 0.537 |
| Mucositis | 0 | 0 | / | 0 | 1 | 0.295 | 0 | 0 | / | 0 | 1 | 0.325 |
| Dysgeusia | 0 | 0 | / | 0 | 0 | / | 0 | 0 | / | 0 | 0 | / |
| Limb numbness | 1 | 0 | 0.301 | 1 | 1 | 0.949 | 1 | 1 | 0.950 | 1 | 0 | 0.306 |
| Abbreviations: ALT, alanine aminotransferase; AST, aspartate aminotransferase‌; FOLFOX, fluorouracil, leucovorin and oxaliplatin; SOX, S-1 and oxaliplatin. | | | | | | | | | | | | |
