# Supplementary material for: High Loss of Adipose Tissue During Neoadjuvant Chemotherapy Predicts Poor Prognosis in Patients With Gastric Cancer
Source: J Cachexia Sarcopenia Muscle. 2025 Oct 30;16(6):e70107. doi: 10.1002/jcsm.70107 (PMC12575444; doi:10.1002/jcsm.70107)
Supplement: Supplementary file 2 — Data S1: Supporting Information. [file JCSM-16-e70107-s002.docx]

**Trial protocol**

| **Title** | S-1 plus oxaliplatin compared with fluorouracil, leucovorin plus oxaliplatin as perioperative chemotherapy for locally advanced, resectable gastric cancer (FOCUS): a multi-center, open-labeled, randomized controlled trial |
| --- | --- |
| **Study Site** | China, 12 clinical study centers in total |
| **Study Period** | Recruitment start date: June 2011  Recruitment end date: August 2016  Follow-up period end date: September 2019  Secondary analysis end date: October 2024 |
| **Population** | Primary tumor penetrated serosa or invaded adjacent structures with or without metastatic lymph nodes (T4N+/-) according to the American Joint Committee on Cancer and Union for International Cancer Control (AJCC-UICC) TNM classification for carcinoma of stomach (7th edition) |
| **Endpoints** | **Primary Endpoint:**   - To demonstrate that, for patients with locally advanced gastric cancer (cT4NanyM0), the 3-year overall survival rate of perioperative chemotherapy of S-1 plus oxaliplatin was non-inferior to that of fluorouracil, leucovorin plus oxaliplatin.   **Secondary Endpoints:**   - To demonstrate that, for patients with locally advanced gastric cancer (cT4NanyM0), the 3-year Progression-free survival rate of perioperative chemotherapy of S-1 plus oxaliplatin was non-inferior to that of fluorouracil, leucovorin plus oxaliplatin. - To compare the safety of perioperative SOX versus FOLFOX regimen. |
| **Trial design** | Randomized, open-label, multicenter, non-inferiority study |
| **Sample size** | The assumed 3-year OS of advanced gastric cancer patients who received perioperative chemotherapy of FOLFOX was about 32%, and the prespecified non-inferiority margin was -8% for SOX. With a 10% dropout, 583 patients are required to reach a power of 80%, at a two-sided type 1 error of 0.05. The OS and PFS were estimated by Kaplan-Meier analysis. To prove non-inferiority, we used the difference of 3-year OS between the two groups, calculated the one-sided 95% confidence interval (CI) using the Kaplan-Meier estimates and Greenwood’s estimates of the corresponding variance, and established whether it was greater than the prespecified non-inferiority margin of -8%. |
| **Inclusion and exclusion criteria** | **Inclusion criteria:**  (1) Histopathologically confirmed gastric adenocarcinoma  (2) Primary tumor penetrated serosa or invaded adjacent structures with or without metastatic lymph nodes (T4N+/-) according to the American Joint Committee on Cancer and Union for International Cancer Control (AJCC-UICC) TNM classification for carcinoma of stomach (7th edition)  (3) Ambulatory males or females  (4) Aged 18-80 years old  (5) Eastern Cooperative Oncology Group (ECOG) score 0-2  (6) Life expectancy more than 3 months  (7) Sufficient bone marrow function (white blood cell count: 3.5×109/L~12×109/L; platelet count >100×109/L), liver function (total bilirubin <1.5×upper limit of normal [ULN]; alanine aminotransferase or aspartate amino transferase <2.5×ULN), renal function (calculated glomerular filtration rate >80ml/min or serum creatinine < 1.5×ULN), cardiac function (ejection fraction > 50% by echocardiograph).  **Exclusion criteria:**  (1) Distant metastasis according to the AJCC Cancer Staging Manual (7th Edition);  (2) History of major stomach surgery;  (3) Previous cytotoxic chemotherapy, radiotherapy, target therapy or immunotherapy for any tumor;  (4) History of another malignancy except for cured basal cell carcinoma of skin and cured carcinoma in-situ of uterine cervix;  (5) Massive gastrointestinal hemorrhage and/or gastric outlet obstruction caused by tumor;  (6) Continuous systematic administration of corticosteroids;  (7) History of angina, myocardial infarction within 6 months or other serious uncontrolled concomitant diseases. |
| **Study procedure** | **Screening Period：**Select visit must be completed within 14 days before the written informed consent. All subjects were enrolled according to the inclusion and exclusion criteria. A written informed consent must be obtained before study treatment. Select visit included:   - Tumor staging and evaluation investigation mainly included endoscopy, computed tomography (CT). Endoscopic ultrasound (EUS), magnetic resonance imaging (MRI) were undertaken according to local practice. - Demographic data, medical history (including GC and related treatment history, and concomitant diseases and treatments), physical examination, pregnancy test (if necessary). - ECOG score, body height and body weight, hematology and blood biochemistry tests. - If the existing hematology and/or blood biochemistry tests were more than 7 days before the treatment initiation, such laboratory tests should be repeated within 1 week prior to the initiation of chemotherapy   **Treatment period:** All enrolled subjects received perioperative chemotherapy, and the regimens were as follows:  For SOX group:   - Oxaliplatin：administered as a 2-h intravenous infusion 130 mg/m^2^ on day 1. - S-1: given orally twice daily for 2 weeks followed by a 7-day rest period. The dose of S-1 was 80 mg/day for body surface area (BSA) <1.25 m^2^. 100 mg/day for BSA ≥1.25 to <1.5 m^2^, and 120 mg/day for BSA ≥1.5 m^2^).   For FOLFOX group:   - Oxaliplatin：administered as a 2-h intravenous infusion 130 mg/m^2^ on day 1. - Leucovorin 400 mg/m^2^ - A bolus of 5-FU 400 mg/m^2^ on day 1, followed by a 46-h infusion of 5-FU at 2400 mg/m^2^.   • The above regimens were repeated every 3 weeks.  • A total of 6 cycles of perioperative chemotherapy were scheduled for enrolled patients. 2-4 cycles before surgery followed by 2-4 cycles after surgery.  • Surgery was scheduled within 2 weeks after completion of the last cycle of preoperative chemotherapy.  • Postoperative chemotherapy was to be started within 4-6 weeks after surgery.  **Follow up:**  After completion of the treatment, enrolled patients were followed up every 3-6 months from 1-2 years, 6-12 months from 3-5 years, and then annually after 5 years. The follow-up included complete blood counts, chemistry profile, tumor markers, and radiological examinations. Gastrointestinal endoscopy was conducted annually. Whether the tumor recurred or progressed was determined by radiological findings or tissue biopsy if it was feasible. Cause of death and sites of recurrence or progression were assessed and recorded by investigators.  **Clinical response evaluation:**  The clinical response is evaluated mainly by computed tomography (CT).  • The preoperative imaging assessment will be performed every 2 cycles of preoperative therapy and before surgery.  • After finishing all scheduled treatment, subsequent imaging assessment will be performed every 12 weeks (±7days) for the first 2 years and every 24 weeks (±7days) thereafter.  **Patient who discontinues the study treatment should undergo the scheduled imaging assessment until initiating new anti-tumor therapy, withdrawal the informed consent, death or the completion of the study. Patients who were confirmed progression disease or recurrence during treatment must discontinue the study drug. The following treatment were determined by investigators.**  **Safety assessment：**  All adverse events (AE) should be recorded during the study. The relationship between AE and the study regimen should be analyzed. The AE should be recorded and assessed until 90 days after the last dose of study regimen. Adverse events during chemotherapy were graded according to National Cancer Institute Common Terminology Criteria for  Adverse Events (CTCAE) 3.0 Any postoperative complications were evaluated within 30 days after surgery and graded using the Clavien-Dindo criteria. |
| **Principle investigator** | Jiren Yu |
